# Contactin 1 (CNTN1) expression associates with regional lymph node metastasis and is a novel predictor of prognosis in patients with oral squamous cell carcinoma

**DOI:** 10.3892/mmr.2012.910

**Published:** 2012-05-09

**Authors:** HE-MING WU, WEI CAO, DONGXIA YE, GUO-XIN REN, YU-NONG WU, WEI GUO

**Affiliations:** 1 Shanghai Key Laboratory of Stomatology, Department of Oral and Maxillofacial - Head and Neck Oncology, Ninth People's Hospital, School of Medicine, Shanghai Jiao Tong University, Shanghai 200011; 2 Institute of Stomatology, Department of Oral and Maxillofacial Surgery, Nanjing Medical University, Nanjing, Jiangsu 210029, P.R. China

**Keywords:** contactin 1, oral squamous cell carcinoma, prognosis, invasion

## Abstract

The contactin 1 (CNTN1) gene exerts oncogene-like activities and its expression has been linked to several human malignancies. In this study, a possible association between CNTN1 expression and clinicopathological parameters and clinical outcomes in patients with oral squamous cell carcinoma (OSCC) was examined. CNTN1 protein expression was evaluated by immunohistochemistry in OSCC tissues of 45 patients. For the immunohistochemical assessment of CNTN1 expression, the cytoplasmic staining labeling index was analyzed using a semiquantitative score. The association between CNTN1 protein levels and clinicopathological factors was analyzed using the Mann-Whitney U test for categorical variables and the Kruskal-Wallis test for continuous variables. The effects of CNTN1 expression on overall and disease-free survival were assessed by using univariate survival analysis. The transcript levels of CNTN1 were detected in OSCC cell lines. In addition, specific siRNA against CNTN1 was applied to investigate the effect exerted by CNTN1 ablation on OSCC cell lines by proliferation and invasion assays *in vitro*. During follow-up, 16 patients (35.56%) had succumbed to OSCC; the median follow-up of patients was 5.0 years (range, 0.2–8.3). A high expression of CNTN1 was markedly associated with the regional lymph node metastasis of patients with OSCC (P=0.006). CNTN1 expression was significantly associated with overall survival of patients with OSCC (P=0.032; log-rank test) and disease-free survival of patients with OSCC (P=0.038; log-rank test). In addition, CNTN1 ablation notably suppressed the invasion potential of OSCC cell lines, but there was no significant change in the proliferation of OSCC cell lines by CNTN1 knockdown *in vitro*. The study supports CNTN1 as a novel predictor of regional lymph node metastasis in patients with OSCC and a prognostic marker for OSCC in patients.

## Introduction

Oral squamous cell carcinoma (OSCC) accounts for more than 90% of oral cavity tumors. With approximately 8000 mortalities per year nationally, it constitutes approximately 3% of all cancer cases in the United States and is one of the six most frequent types of cancer worldwide (1). Although tobacco and alcohol are regarded as primary risk factors, it is clear that genetic and epigenetic factors contribute to this cancer (2). On this basis, novel molecular markers involved in OSCC development and progress should be investigated.

The protein encoded by the CNTN1 gene is a member of the immunoglobulin superfamily, which includes N-CAM, L1 and Nr-CAM. CNTN1 is a glycosylphosphatidylinositol (GPI)-anchored neuronal membrane protein that functions as a cell adhesion molecule (3). It mediates cell surface interactions during nervous system development, involving the formation of paranodal axo-glial junctions in myelinated peripheral nerves and signaling between axons and myelinating glial cells via its association with CNTNAP1. In addition, as a ligand of Notch1, CNTN1 promotes Notch1 activation which is involved in oligodendrocyte generation through the released notch intracellular domain (NICD) and subsequent translocation to the nucleus (4). However, it is becoming increasingly evident that certain members of this immunoglobulin superfamily facilitate the motility, invasion and metastasis of cancer (5,6). Additionally, the location of CNTN1 in the 12q11-q12 chromosomal region, which is a breakpoint region in several types of cancer, suggests that CNCT1 is involved in tumor formation or progression.

Notably, *in vitro* silencing of CNTN1 expression may inhibit the invasive and metastatic ability of lung adenocarcinoma cells (7). Furthermore, VEGF-C/Flt-4-mediated invasion and metastasis of cancer cells were found to be through the upregulation of the neural cell adhesion molecule CNTN1 which activated the Src-p38 MAPK-C/EBP-dependent pathway (8). In view of its malignant phenotype-promoting activities in cancer cells and its growth-promoting abilities in neural cells, this study investigated the possibility of CNTN1 as a prognostic marker for patients with OSCC and the association between CNTN1 expression and metastasis of OSCC *in vivo*. CNTN1 protein levels were evaluated in 45 primary OSCC specimens by immunohistochemistry. Our study demonstrates that CNTN1 protein level was markedly associated with lymph node metastasis of patients with OSCC (P=0.006). CNTN1 expression was significantly associated with overall survival of patients with OSCC (P=0.032; log-rank) and disease-free survival of patients with OSCC (P=0.038; log-rank). *In vitro* results revealed that CNTN1 ablation was able to inhibit the invasion potential of OSCC cells, but not proliferation of OSCC cells. We conclude that CNTN1 is a novel and powerful factor for the metastasis and prognosis of OSCC patients.

## Patients and methods

### Patients and specimens

Patients (n=45) with stage I to IV OSCC who underwent radical surgery at the Department of Oral and Maxillofacial Surgery, Shanghai Ninth People's Hospital, Shanghai Jiao Tong University School of Medicine, Shanghai, China between January 2002 and December 2002, who had not undergone radio-or chemotherapy, were enrolled into this prospective study. All of the tumors were classified according to the International Union Against Cancer (UICC) tumor/lymph node/metastasis (TNM) classification system (9). Histological diagnoses of OSCC were made according to the criteria of the World Health Organization (WHO) for the histological typing of cancer (10). Patients were biopsied and histopathologically examined at the Ninth People's Hospital, Shanghai Jiao Tong University School of Medicine, Shanghai, China. Patients were prospectively evaluated (chest X-ray or thoracic CT scan, abdominal sonography or CT scan or MRI and serum chemistry) every 3 months for the first 2 years after surgery, every 6 months for the following 3 years and annually thereafter. This study was approved by the ethics committee of Shanghai Ninth People's Hospital. Informed consent was obtained from each patient. A total of 45 patients with follow-up periods up to 8.3 years were included in the study. Annual follow-up data were retrieved from the medical records. The specimens were fixed in 10%-buffered formalin and embedded in paraffin wax. Paraffin blocks were sectioned into 4 μm slices.

### Cell lines

The human HNSCC cell lines Tca, Tca-M, Tb, Tca/CDDP (kindly provided by the Shanghai Ninth People's Hospital, Shanghai, China), TSCC (kindly provided by Wuhan University, School of Medicine, China), OSC-4, NB and NT (kindly provided by Kochi University, School of Medicine, Japan) were cultured in RPMI-1640 medium (Gibco-BRL, Carlsbad, CA, USA) supplemented with 10% heat-inactivated fetal bovine serum (FBS; Gibco-BRL), penicillin (100 U/ml) and streptomycin (100 μg/ml) at 37°C in a humidified 5% CO_2_ atmosphere. CAL27 (American Type Culture Collection, Manassas, VA, USA) was cultured in Dulbecco's modified Eagle is medium (DMEM; Gibco BRL) supplemented with 10% heat-inactivated FBS (Gibco BRL), penicillin (100 U/ml) and streptomycin (100 μg/ml) at 37°C in a humidified 5% CO_2_ atmosphere.

### Immunohistochemistry

The avidin-biotin complex (ABC) technique was performed using a Vectastain Elite ABC kit (Vector Laboratories, Inc., Burlingame, CA, USA). Briefly, paraffin-embedded tissue sections were dewaxed and rehydrated using xylene and a series of graded alcohols. To determine antigenicity, slides were steamed with 10 mmol/l citrate buffer (pH 6.0; DAKO/Cytomation, Glostrup, Denmark) for 20 min. Endogenous peroxidase activity was quenched by immersing the slides in 3% hydrogen peroxide in double-distilled water for 20 min. Tissue sections were blocked with 10% normal horse serum for 30 min at room temperature. The slides were then incubated with monoclonal anti-CNTN1 antibody at 1:100 dilution (Santa Cruz Biotechnology, Inc., Santa Cruz, CA, USA) at 4°C overnight. Each section was treated with biotinylated-secondary antibody for 30 min at room temperature. Diaminobenzidine was used as the chromogen for the immunoperoxidase reaction and the slides were counterstained with Mayer's hematoxylin (DAKO/Cytomation). Sections were thoroughly washed, glass covered and analysed by light microscopy, using a magnification of up to ×400. For the immunohistochemical assessment of CNTN1 expression, the frequency of cytoplasmic staining was evaluated using a semiquantitative score: 0–1, from negativity to positivity in <50% (low expression); 2, positivity in >50% (high expression). The score of each lesion was the average of the indices generated by two observers (W.C. and H.M.W.) blinded to the clinical information. The differences between the two observers were <10% in almost all cases.

### Reverse transcription-polymerase chain reaction (RT-PCR) analysis

High quality total RNA (2 μg) was directly processed to cDNA using the reverse transcription kit (Promega, Madison, WI, USA), following the manufacturer's instructions, in a total volume of 25 μl. The primer sequences used were: CNTN1, forward: 5′-CAACAAAACCATATCCTGCTGA-3′; reverse: 5′-AGATCACTGCCTATGTCCACCT-3′; β-actin, forward: 5′-TCACCCACACTGTGCCCATCTACGA-3′; reverse: 5′-CAGCGGAACCGCTCATTGCCAATGG-3′; Each primer was added at a final concentration of 0.5 μM to a 15 μl reaction mixture in PCR buffer, containing 1 μl cDNA, 0.25 mM of each dNTPs, 1.5 mM MgCl_2_ and 2.5 units Taq DNA polymerase. An initial denaturation was conducted for 5 min at 94°C and 35 cycles were performed with the following PCR program: denaturation at 94°C for 30 sec, annealing at 45 for 60°C for CNTN1 for 30 sec and 55°C for β-actin for 30 sec, elongation at 72°C for 30 sec, followed by a final extension of 5 min at 72°C. Ethidium bromide-stained bands were visualized by UV transillumination and the fluorescence intensity was quantified using the FR-200 system (Shanghai FURI Science and Technology Co., Ltd., Shanghai, China). RT-PCR data were from at least three independent experiments.

### Small interfering RNA (siRNA) knockdown

Two siRNAs against CNTN1 were designed and chemically synthesized (Shanghai GenePharma Co., Ltd., Shanghai, China). The siRNA which had a greater silencing effect was selected for further study, with the following sequence: CNTN1-siRNA_1697: 5′-GGUCCUUCAAUGGCUAUGUTT-3′ and 5′-ACA UAGCAUUGAAGGACCTT-3′ for nucleotides 1697–1718. In addition, a negative control, siRNA_NC; 5′-UUCUCCGAACGUGUCACGUTT-3′ and 5′-ACG UGACACGUUCGGAGAATT-3′ was also synthesized. The *in vitro* transient transfection was performed using Lipofectamine 2000 (Invitrogen, Carlsbad, CA, USA) according to the manufacturer's instructions.

### Proliferation assay

Proliferation assays were performed to analyze the proliferation potential of transient-transfected si-RNA_1697, siRNA_NC and Lipofectamine 2000 only into cells by using the Cell-Counting kit (CCK)-8 (Dojindo, Kumamoto, Japan). The cells were harvested and plated onto 96-well plates at 1×10^3^ cells per well and maintained at 37°C in a humidified incubator. At the indicated times, 10 μl of the CCK-8 solution were added into the triplicate wells and incubated for 1 h and the absorbance at 450 nm was measured to calculate the number of vital cells in each well. Cells were performed in triplicate and the mean [± standard deviation (SD)] optical density (OD) was reported.

### Invasion assays

A total of 1×10^5^ various cells in 750 μl serum-free RPMI-1640 medium were plated onto a BD BioCoat™ Matrigel™ Invasion Chamber (8 μm pore size; BD Biosciences, Franklin Lakes, NJ, USA) and the lower chamber was immediately filled with 750 μl of RPMI-1640 medium with 10% FBS as a chemoattractant. After 48 h of incubation in a humidified atmosphere containing 5% CO_2_ at 37°C, the non-invading cells were removed from the upper surface of the membrane by a cotton swab and the membranes were then fixed with methanol and stained with 0.5% crystal violet. Invading cells were captured and counted in five random non-overlapping fields under a light microscope (magnification, ×100).

### Statistical analysis

The associations between CNTN1 expression status and clinicopathological parameters were analyzed using the Mann-Whitney U test and the Kruskal-Wallis test for categorical variables. The probability of overall survival and disease-free survival by CNTN1 protein expression was determined using the Kaplan-Meier method. Paired t-test was used for analysis of the *in vitro* studies. Analyses were conducted using SAS 9.1.3 software. The tests were two-sided, and P<0.05 was considered to indicate a statistically significant difference.

## Results

### Correlation between CNTN1 expression and patient characteristics

**Figure 1:**
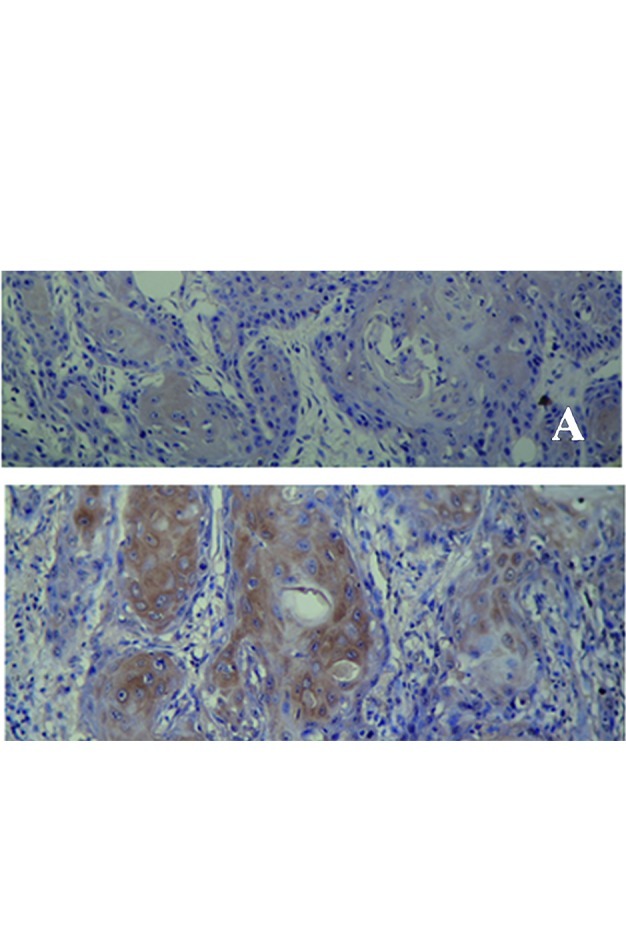
Expression of CNTN1 in OSCC. CNTN1 expression was detected within the cytoplasm of OSCC cells. (A) Low staining of CNTN1 in OSCC CNTN1. (B) High staining of CNTN1 expression in OSCC. (A and B): Original magnification, ×100. CNTN1, contactin 1; OSCC, oral squamous cell carcinoma.

To identify the association between CNTN1 expression and clinicopathological factors, the expression of CNTN1 was analyzed in 45 primary OSCCs by immunohistochemistry. Positive lesions demonstrated clearly membrane or cytoplasmic localization of the CNTN1 protein (Fig. 1), which is consistent with its function in cells. Distribution of the CNTN1 expression status and associations with general clinicopathological parameters are shown in Table I. Of 45 patients enrolled in this study, 12 (26.7%) patients had regional lymph node metastasis to a certain extent. Furthermore, 9 (75%) of 12 (26.7%) patients with regional lymph node metastasis revealed a high expression level of CNTN1, whereas 22 (66.7%) of 33 (73.33%) patients without regional lymph node metastasis revealed a low expression level of CNTN1. A significantly high expression level of CNTN1 was found to be markedly association with patients who had regional lymph node metastasis. Additionally, no statistically significant correlation was found between CNTN1 protein level and other clinicopathological factors, including age (P=0.063), gender (P=0.064), clinical TNM classification (P=0.069), pathological grade (P=0.527) and tumor recurrence (P=0.640).

### CNTN1 expression and patient survival

**Figure 2:**
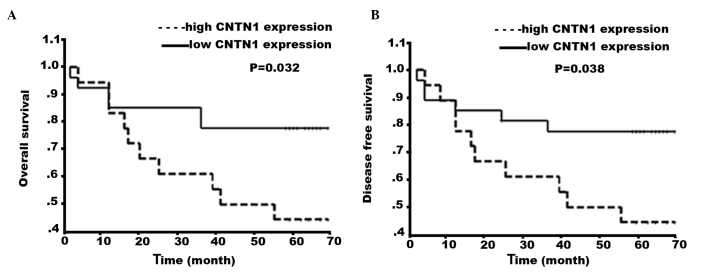
Survival rate according to tumor CNTN1 staining. (A) Overall survival rate (n=45, P=0.032), (B) disease-free survival rate (n=45, P=0.038) of OSCC patients. Solid line, patients with reduced or no expression (levels 1 and 0) of CNTN-1; dotted line, patients with a high expression of CNTN-1 (levels 2). P-value was determined by a two-sided log-rank test. CNTN1, contactin 1.

To determine whether CNTN1 protein expression is a prognostic marker for patients with OSCC, the overall and the disease-free survival of OSCC were calculated using the log-rank test and curves were constructed using the Kaplan-Meier method (Fig. 2). The median follow-up of patients was 5.0 years (range, 0.2–8.3). During the follow-up of 45 patients, 16 patients (35.56%) succumbed to OSCC. Of these 16 patients, 6 patients (37.5%) demonstrated a low protein level of CNTN1, while 10 patients (62.5%) demonstrated a high protein level of CNTN1. The survival of patients with OSCC was calculated from the time of radical surgery until the end of the follow-up. The disease-free survival rate at 3 and 5 years after radical surgery for the whole cohort of patients was 71.11 and 64.44%, respectively. Survival analysis revealed that CNTN1 expression was significantly associated with overall survival of patients with OSCC (P=0.032; log-rank) and disease-free survival of patients with OSCC (P=0.038; log-rank).

### Effect of CNTN1 ablation on cell proliferation of OSCC cell lines

**Figure 3:**
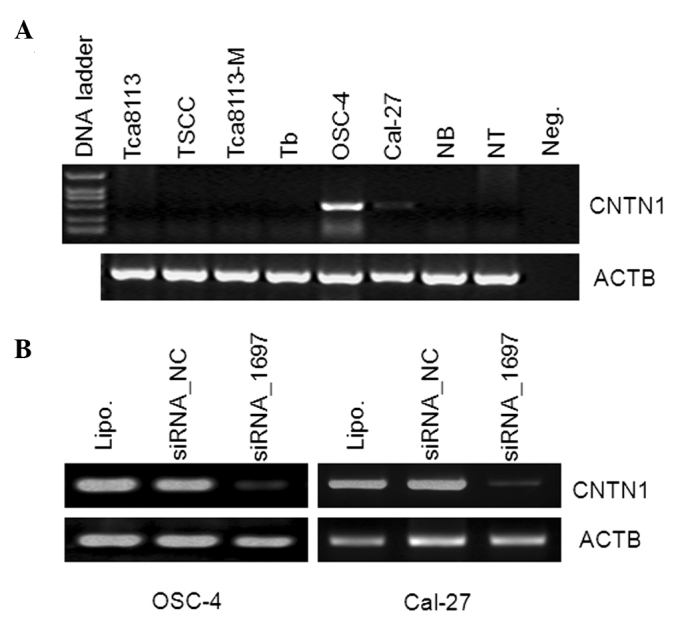
CNTN1 expression in different oral squamous cell carcinoma cell lines and suppression of CNTN-1 expression by transient-transfected siRNA. (A) Expression of CNTN1 mRNA in different oral squamous cell carcinoma cell lines. OSC-4 and Cal-27 cells were highly expressed. (B) Suppression of CNTN1 expression by transient transfection-targeted CNTN1 siRNA (left panel OSC-4; right panel, Cal-27). Lipo., Lipofectamine 2000. CNTN1, contactin 1; siRNA, small interfering RNA.

**Figure 4:**
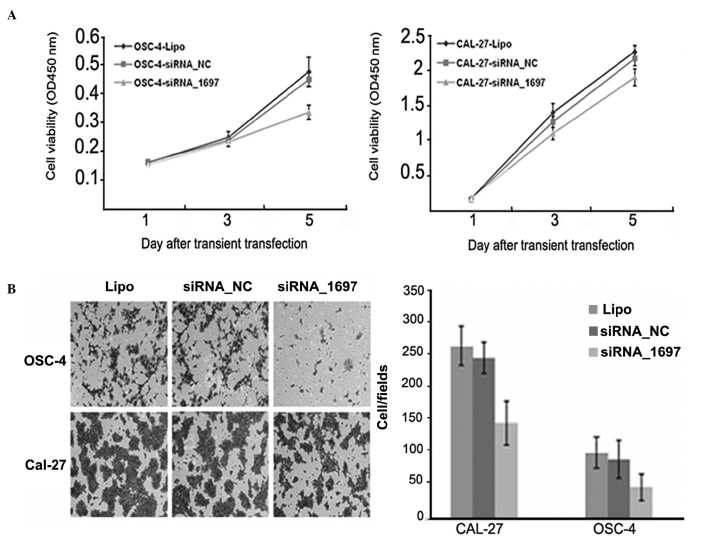
Proliferation and invasive ability of OSCC cells after suppression of CNTN1 expression. (A) Proliferation of OSCC cells was not markedly inhibited after CNTN1 siRNA, compared to cells transfected with siRNA_NC control (left panel, OSCC-4; right panel, Cal-27 cells). (B) Suppression of CNTN1 expression decreased the invasive ability of OSCC cells. Left panel, *in vitro* invasion assays; right panel, number of cells invading through the transwell membrane in OSC-4 transfected with siRNA_1697 and Cal-27 transfected with siRNA_1697 were significantly lower than those transfected with siRNA_NC, respectively (P<0.01, two-tailed Student's t-test). Lipo, Lipofectamine 2000; OSCC, oral squamous cell carcinoma; CNTN1, contactin 1; siRNA, small interfering RNA.

To evaluate whether CNTN1 promotes malignant phenotypes of OSCC cells, we firstly assessed the effect of CNTN1 ablation on cell proliferation of OSCC cell lines. Based on the CNTN1 expression pattern in OSCC-derived cell lines (Fig. 3A), we transiently transfected target CNTN1 siRNA, negative control siRNA_NC and Lipofectamine 2000-only to OSC-4 and Cal-27 cells, all of which have endogenous CNTN1 mRNA expression (Fig. 3B). However, our results demonstrated that silencing CNTN1 did not remarkably inhibit the proliferation of OSCC cells compared to cells transfected with siRNA_NC control (Fig. 4A). Furthermore, *in vitro* invasion assays were performed to determine the effect of CNTN1 on cell invasion using a BD BioCoat Matrigel Invasion Chamber. The Matrigel matrix served as a reconstituted basement membrane *in vitro*. Moreover, the number of cells invading through the transwell membrane in OSC-4 transfected with siRNA_1697 and Cal-27 transfected with siRNA_1697 was significantly lower than those transfected with siRNA_NC, respectively (P<0.01) (Fig. 4B).

## Discussion

As one of the most common types of epithelial cancer, the incidence of oral squamous cell carcinoma (OSCC) is the sixth highest worldwide (11). Traditional treatments, including radical surgery, radiotherapy and chemotherapy, have not sufficiently improved the five-year survival rate of patients with this disease in more than two decades. Moreover, the development of OSCC is evolutionary and characterized by multistep carcinogenic processes, in which the activation of oncogenes and inactivation of tumor suppressor genes are key features leading to OSCC progression. Considering these factors, the identification of useful predictors or targets of OSCC for diagnosis, therapy and prognosis is promising. Although numerous studies have been conducted, few useful molecular predictors or targets for OSCC have been identified.

CNTN1 is a novel member of the contactin subgroup of the immunoglobulin superfamily which also includes contactin-2, 5 and 6. The well-known role of these proteins and ligands is the repulsive guidance of nerve axons, regulating neurite extension in a mouse neuroblastoma cell line and primary hippocampal neurons (12–14). In addition, mutations in the CNTN1 gene causing a familial type of lethal congenital myopathy have been reported (15). In addition to its regulatory role in the nervous system, CNTN1 functions as a glycosylphosphatidylinositol anchor neural cell adhesion molecule (NCAM), which is involved in tumor cell adhesion, invasion and metastasis (16–18). CNTN1 was first described as a metastasis-promoting oncogenic protein by Su *et al* (7,8). Suppression of CNTN1 expression abolished the ability of lung cancer cells to invade and metastasize by activating RhoA, but not Cdc42 or Rac1, suggesting that CNTN1 is a key regulator of invasion and metastasis in lung adenocarcinoma (7). Considering the particularity of OSCC in epidemiology, which is notably different from other cancer types, as well as the different molecule signatures in invasion and metastasis, in this study, we investigated whether CNTN1 is a major factor promoting OSCC progression and metastasis. Experimental results demonstrated that CNTN1 expression is markedly associated with regional lymph node metastasis (P=0.006) in patients with OSCC and silencing CNTN1 decreased the invasion potential of OSCC cells, confirming that CNTN1 is a powerful factor involved in invasion and metastasis in OSCC. Notably, CNTN1 ablation exerted little effect on the proliferation of OSCC cells, indicating that CNTN1 promotes malignant phenotypes of OSCC by exclusively activating the metastastic potential.

Our data demonstrated that CNTN1 expression was significantly associated with the overall survival of patients with OSCC (P=0.032; log-rank test) and disease-free survival of patients with OSCC (P=0.038; log-rank test) by univariate analysis. Thus, CNTN1 may be a novel predictor of clinical outcome for patients with OSCC.

## Figures and Tables

**Table I:** Correlation between CNTN1 expression and patient characteristics.

	CNTN1 expression (No. of cases)		
		
Characteristics	Low	High	P-value
Age at diagnosis			
<60 years	20	8	0.063
≥60 years	7	10	
Gender			
Female	8	11	0.064
Male	19	7	
TNM classification			
I or II	16	3	0.069
III or IV	11	15	
Regional metastasis			
With	3	9	0.006
Without	24	9	
Recurrence			
With	3	1	0.640
Without	24	17	
Histological grading			
I	11	5	0.527
II	16	13	
III	0	0	

CNTN1, contactin 1.

## References

[1] Jemal A, Siegel R, Ward E (2009). Cancer Statistics. CA Cancer J Clin.

[2] Warnakulasuriya S (2009). Global epidemiology of oral and oropharyngeal cancer. Oral Oncol.

[3] Falk J, Bonnon C, Girault JA, Faivre-Sarrailh C (2002). F3/contactin, a neuronal cell adhesion molecule implicated in axogenesis and myelination. Biol Cell.

[4] Hu QD, Ang BT, Karsak M (2003). F3/contactin acts as a functional ligand for Notch during oligodendrocyte maturation. Cell.

[5] Prag S, Lepekin EA, Kolkova K (2002). NCAM regulates cell motility. J Cell Sci.

[6] Shtutman M, Levina E, Ohouo P, Baig M, Roninson IB (2006). Cell adhesion molecule L1 disrupts E-cadherin-containing adherens junctions and increases scattering and motility of MCF7 breast carcinoma cells. Cancer Res.

[7] Su JL, Yang CY, Shih JY (2006). Knockdown of contactin-1 expression suppresses invasion and metastasis of lung adenocarcinoma. Cancer Res.

[8] Su JL, Yang PC, Shih JY (2006). The VEGF-C/Flt-4 axis promotes invasion and metastasis of cancer cells. Cancer Cell.

[9] Sobin LH, Fleming ID (1997). TNM Classification of Malignant Tumors, fifth edition (1997). Union Internationale Contre le Cancer and the American Joint Committee on Cancer. Cancer.

[10] Pindborg JJ, Reichart PA, Smith CJ (1997). Histological Typing of Cancer and Precancer of the Oral Mucosa.

[11] Mignogna MD, Fedele S, Lo Russo L (2004). The World Cancer Report and the burden of oral cancer. Eur J Cancer Prev.

[12] Reid RA, Bronson DD, Young KM, Hemperly JJ (1994). Identification and characterization of the human cell adhesion molecule contactin. Brain Res Mol Brain Res.

[13] Mikami T, Yasunaga D, Kitagawa H (2009). Contactin-1 is a functional receptor for neuroregulatory chondroitin sulfate-E. J Biol Chem.

[14] Eckerich C, Zapf S, Ulbricht U (2006). Contactin is expressed in human astrocytic gliomas and mediates repulsive effects. Glia.

[15] Compton AG, Albrecht DE, Seto JT (2008). Mutations in contactin-1, a neural adhesion and neuromuscular junction protein, cause a familial form of lethal congenital myopathy. Am J Hum Genet.

[16] Lehembre F, Yilmaz M, Wicki A (2008). NCAM-induced focal adhesion assembly: a functional switch upon loss of E-cadherin. EMBO J.

[17] Van Kilsdonk JW, Wilting RH, Bergers M (2008). Attenuation of melanoma invasion by a secreted variant of activated leukocyte cell adhesion molecule. Cancer Res.

[18] Gavert N, Sheffer M, Raveh S (2007). Expression of L1-CAM and ADAM10 in human colon cancer cells induces metastasis. Cancer Res.

